# Negotiated Ventilation: Conceptualising Interprofessional Decision Work in Intensive Care—An Ethnographic Study

**DOI:** 10.1111/jan.70685

**Published:** 2026-07-21

**Authors:** Andreas Küpper, Marcel Schmucker, Laura Hahn, Astrid Elsbernd, Simon Kitto, Cornelia Mahler

**Affiliations:** ^1^ Institute for Health Care and Nursing Sciences, Faculty of Social Work, Education and Nursing Sciences Esslingen University of Applied Sciences Esslingen Germany; ^2^ Department of Nursing Science University Hospital Tübingen Tübingen Germany; ^3^ Institute of Learning Mohammed Bin Rashid University of Medicine and Health Sciences Dubai UAE

## Abstract

**Aim:**

To conceptualise how ventilation‐related decisions are accomplished as interprofessional work in intensive care practice, moving beyond approaches that treat decision‐making as discrete events.

**Design:**

Ethnography.

**Methods:**

Data were generated through 97 h of participant observation and 17 episodic interviews with nurses (*n* = 12) and physicians (*n* = 5) in two adult intensive care units of a German university hospital. Data collection and analysis proceeded iteratively between December 2023 and July 2025. Analysis was guided by the Qualitative Analysis Guide of Leuven.

**Results/Findings:**

Decision‐making was found to be an ongoing, socially organised form of work unfolding across the patient's illness trajectory. The analysis differentiates between directional and adjustment decisions and identifies four interrelated domains of decision work: orienting, involving, negotiating and implementing. The findings highlight (1) the temporal and iterative organisation of decision work, (2) involving in organising participation and access to influence, (3) the significance of non‐negotiation practices in stabilising decisions and (4) implementing as a constitutive and relational dimension through which decisions are enacted in practice.

**Conclusions:**

Viewing ventilation‐related decision‐making as interprofessional decision work shifts attention from discrete choices to the ongoing accomplishment of decisions in practice. This perspective makes visible how decision‐making is shaped through distributed and practice‐based contributions, with nurses playing a central role, particularly through implementing and adjustment work that remains underrepresented in cognitive models of decision‐making.

**Implications:**

The conceptualisation of ventilation‐related decision work supports more explicit recognition of nursing contributions in interprofessional collaboration, particularly in shaping decisions through adjustment and implementing practices and may inform approaches to organising decision‐making in complex care settings.

**Reporting Method:**

This study was reported in accordance with the Standards for Reporting Qualitative Research (SRQR).

**Patient or Public Contribution:**

This study did not include patient or public involvement in its design, conduct, or reporting.

## What Does This Paper Contribute to the Wider Global Clinical Community?

- Ventilation‐related decision‐making is conceptualised as ongoing interprofessional decision work unfolding across the illness trajectory, rather than as discrete decision events.
- Interprofessional decision work is accomplished not only through information sharing but through social practices of involving, non‐negotiation and implementation that organise participation and influence.
- This conceptualisation enables analysis of interprofessional dynamics and professional contributions, particularly those of nurses, that remain underrepresented in cognitive models of decision‐making.

**Abbreviations**
ECLS: extracorporeal life support
ECMO: extracorporeal membrane oxygenation
ICU: intensive care unit
QUAGOL: Qualitative Analysis Guide of Leuven

## 1 Introduction

The care environment in intensive care units (ICUs) is characterised by significant dynamism due to the rapidly changing and critical health conditions of patients. Consequently, clinicians are frequently required to make decisions of considerable importance and complexity (Matsumoto et al. 2025). These decisions are rarely made by individuals alone but are typically worked out within interprofessional constellations, where multiple actors contribute to and influence decision‐making processes (Bouchez et al. 2024). As such, decision‐making in ICUs is inherently embedded in social processes (Bruch and Feinberg 2017). Interprofessional collaboration is widely promoted as a means to improve the quality of clinical decision‐making (Bouchez et al. 2024; Michalsen et al. 2019). However, empirical studies continue to describe decision‐making processes in ICUs as unevenly distributed, often shaped by medical dominance and unequal access to different professional perspectives (Alexanian et al. 2015; Caronia et al. 2020; Coombs and Ersser 2004; Küpper, Schmucker, Hahn, et al. 2026; Manias and Street 2001). Yet, these relationships are not static but have evolved beyond traditional role patterns, reflecting increasingly complex and fluid forms of interprofessional collaboration (Carmel 2006; Xyrichis et al. 2017).

Despite this recognition of the social and interprofessional nature of clinical decision‐making, existing conceptualisations predominantly focus on cognitive processes (Nibbelink and Brewer 2018). This also applies to approaches that explicitly address collaborative aspects of decision‐making, such as collaborative clinical reasoning (Kiesewetter et al. 2017) or distributed cognition (Wilson et al. 2022), which provide valuable insights into how decisions are cognitively processed. While other approaches explicitly consider interprofessional interaction or negotiation, they either focus on structural dimensions of decision‐making (DeKeyser Ganz et al. 2016) or on singular, far‐reaching decisions, such as end‐of‐life discussions (Flannery et al. 2020; Long‐Sutehall et al. 2011), while leaving the ongoing organisation of decision‐making in everyday practice largely unexamined. Thus, although these conceptualisations are valuable for understanding specific aspects of decision‐making, these approaches offer limited analytical access to how decisions are organised and accomplished as situated work within the social complexities of interprofessional clinical practice in ICUs.

The life‐sustaining intervention of mechanical ventilation is a defining feature of modern intensive care (Sterr et al. 2026) and a central focus of interprofessional decision‐making involving technical devices in ICU contexts (Küpper, Schmucker, Elsbernd, et al. 2026). Ventilation decisions in ICUs involve a wide range of different choices (Murali et al. 2024), including a continuous stream of small, situation‐specific decisions that shape everyday care (Karlsen et al. 2020). As ICU work is inherently interprofessional in nature (Pun et al. 2022), the provision of safe and high‐quality ventilation care necessarily relies on interprofessional collaboration (Donovan et al. 2018). Professional roles and responsibilities in ventilatory management vary across systems (Murali et al. 2024). Even within similar systems, variations in decisional responsibility have been documented (Rose et al. 2011). These professional responsibilities, however, do not appear to be fixed in everyday practice. Ethnographic ICU research suggests that professional boundaries are situationally reconfigured in everyday practice rather than being fixed allocations of authority (Carmel 2006; Xyrichis et al. 2017). Taken together, these characteristics make ventilation‐related decision‐making a particularly suitable context for examining how clinical decisions are organised and accomplished in interprofessional ICU practice.

Building on this focus, the present study draws on an ethnographic dataset of ventilation‐related decision‐making in ICU practice. In a prior analysis of this dataset, we examined contextual factors shaping interprofessional roles and responsibilities (Küpper, Schmucker, Hahn, et al. 2026). While this analysis provided insight into the conditions under which responsibilities are configured, it did not address how decisions themselves are organised and accomplished over time in interprofessional practice. To interrogate this, the present study draws on the same dataset but develops a distinct analytical perspective by focusing on the accomplishment of decision‐making as interprofessional work. It is informed by an interactionist understanding of clinical practice, using the well‐established concept of negotiated order (Allen 1997; Nugus 2019; Reeves et al. 2009). Drawing on the interactionist tradition rooted in American pragmatism, professional roles, responsibilities and decisions are understood as continuously worked out in interaction rather than fixed in advance (Strauss et al. 1997). Consequently, the focus of analysis is not on decisions as isolated outcomes but on the everyday practices through which they are accomplished. Ventilation‐related decision‐making is thus approached as work: a temporally unfolding, socially organised process through which decisions are worked out in situated interaction among different actors. As such, ventilation‐related decision work is embedded in the trajectory of a patient's critical illness, understood as the evolving course of the patient's condition and its practical management over time (Strauss et al. 1997). This understanding departs from viewing decisions as discrete moments of selecting the best option and instead brings aspects of practice that are otherwise difficult to capture into focus.

The aim of this study is to develop a practice‐oriented conceptualisation of ventilation‐related decision‐making that captures the social organisation of interprofessional decision work. Accordingly, the study addresses the following research question: How is ventilation‐related decision work structured and accomplished in interprofessional intensive care practice?

## 2 Methodology

### 2.1 Qualitative Approach and Paradigm

Consistent with the interactionist perspective outlined in the Introduction, we adopted an ethnographic approach (Hammersley and Atkinson 2019) to address our research aim. Ethnography was chosen as it represents a well‐established approach within interactionist research traditions (Strauss et al. 1997) and is particularly suited to examining how decision work is accomplished in everyday clinical practice under routine conditions (Hammersley and Atkinson 2019). This study is reported in accordance with the Standards for Reporting Qualitative Research (SRQR) (O'Brien et al. 2014).

### 2.2 Researcher Characteristics and Reflexivity

The research team comprised nursing scientists who are registered nurses (A.K., M.S., L.H., A.E., C.M.), several of whom had prior clinical experience in intensive care (A.K., M.S, C.M.). The team was complemented by a medical sociologist with extensive experience in ICU ethnography (S.K.). The first author's (A.K.) clinical background facilitated field access and contextual understanding but may also have influenced interactions across professional boundaries. Reflexive considerations were addressed throughout the research process and are described in the section on trustworthiness.

### 2.3 Study Setting

The study was conducted in two adult intensive care units of a German university hospital providing tertiary care. One unit was a surgical ICU led by anaesthesiology, caring for critically ill surgical patients from all specialties, including all patients requiring extracorporeal membrane oxygenation (ECMO) or extracorporeal life support (ECLS). At the time of the study, the unit comprised approximately 40 beds. The second unit was a medical ICU with approximately 21 beds, providing care for critically ill patients from all internal disciplines and neurology. Advanced organ support was routinely provided, with the exception of extracorporeal life support, which was managed on the surgical ICU. On both units, care was provided by multiprofessional teams consisting of nurses, physicians and allied health professionals; respiratory therapists were not employed at the time of investigation, which is not unusual in German ICUs.

### 2.4 Sampling and Ethical Approval

Sampling followed a purposive and theoretically informed strategy. Observations focused on nurses and physicians, who, based on prior research (Murali et al. 2024; Rose et al. 2011) and early field impressions, were identified as central actors in ventilation‐related decision work in the ICUs under investigation. While observations encompassed interactions involving a wider range of professional groups, interviews were limited to nurses and physicians to allow for an in‐depth examination of their roles and perspectives. To capture variation in experiences and positions within decision work, participants were sampled across different levels of professional experience and hierarchical seniority. Sampling and data collection evolved iteratively alongside ongoing analysis, in line with ethnographic principles (Hammersley and Atkinson 2019). Ethical approval was granted by the Ethics Committee of the University Hospital Tübingen (project number 142/2023B02). All participants received written and verbal information about the study and provided informed consent. Participation was voluntary, and all data were pseudonymised in accordance with data protection regulations.

### 2.5 Data Collection

Data were collected as part of a broader ethnographic project on ventilation‐related decision‐making in intensive care between December 2023 and July 2025 by the first author. Data generation combined participant observation with informal ethnographic field interviews and episodic interviews. In line with the ethnographic approach, participant observation constituted the primary mode of data generation (Hammersley and Atkinson 2019). Interview data were used to complement and contextualise observational cases, supporting the interpretation of observed decision practices. In total, 97 h of observation were conducted across different shifts. Observations initially focused on familiarisation with everyday routines and were progressively narrowed, guided by ongoing analysis, to situations relevant to ventilation‐related decision work. Fieldnotes were expanded into detailed observation protocols shortly after each observation period; no audio or video recordings were made during observations. To complement observational data, 17 episodic interviews (Flick 2022) were conducted. Table 1 provides an aggregated overview of the interview participants' characteristics. To minimise the risk of deductive disclosure, no more detailed participant information is reported. Interviews lasted between 17 and 52 min (mean 36 min), were conducted face‐to‐face or via videoconference, audio‐recorded and transcribed verbatim with the support of an AI‐based transcription tool (aTrain). Data collection and analysis proceeded iteratively, with insights from ongoing analysis informing subsequent observations and interviews, in line with ethnographic principles (Hammersley and Atkinson 2019). Throughout the analytic process, the developing concept of ventilation‐related decision work was continuously refined. Data generation was concluded once subsequent observations and interviews no longer added substantially new aspects to the developing conceptualisation indicating that the analysis had reached a sufficient degree of analytic saturation for the purposes of the present conceptualisation.

**TABLE 1:** Characteristics of participants.

Characteristic	Interview participants (*n* = 17)
Profession	Nurses (*n* = 12); Physicians (*n* = 5)
ICU experience	Early (*n* = 5); Mid‐level (*n* = 5); Extensive (*n* = 7)
Leadership responsibility	Yes (*n* = 5); No (*n* = 12)

### 2.6 Data Analysis

Data analysis was guided by the Qualitative Analysis Guide of Leuven (QUAGOL), a structured analytic approach that builds on core principles of Grounded Theory, such as iterative analysis, constant comparison and the close linking of empirical material and developing concepts (Dierckx de Casterlé et al. 2012, 2021). These principles align well with an interactionist understanding of clinical practice and with ethnographic approaches (Charmaz and Mitchell 2001). The QUAGOL framework was chosen because it supports in‐depth, case‐oriented engagement with complex qualitative material while avoiding premature abstraction (Dierckx de Casterlé et al. 2012, 2021). As QUAGOL was originally developed for interview data, the approach was adapted for use with ethnographic observation protocols. Observation data were first reconstructed into coherent, case‐oriented narratives of decision processes. Cases were defined as bounded episodes of decision work centred on a specific clinical question, identified through iterative analysis of observation protocols. These reconstructions served as the analytical entry point for subsequent interpretation. Following the QUAGOL methodology, analysis emphasised a case‐oriented approach with extensive preparatory analytic work prior to coding. Formal coding was deliberately postponed to allow for in‐depth, case‐oriented interpretation prior to abstraction and was later used to support systematic comparison across cases (Dierckx de Casterlé et al. 2012).

Translation of quotations from German into English was necessary to make the findings accessible to an international audience; however, all analysis and interpretation were conducted in the original language. Translation constitutes an interpretive act rather than a neutral transfer; therefore, particular attention was paid to preserving semantic and interactional nuances. Nonetheless, shifts in meaning, emphasis, or cultural connotations cannot be fully excluded (McKenna 2022). ChatGPT (OpenAI) and DeepL (DeepL SE) were used to assist the translation process. Their use was approached reflexively, acknowledging their potential to standardise or subtly reframe meaning, and all translations were carefully reviewed by the authors.

### 2.7 Trustworthiness

Trustworthiness was informed by Richardson's criteria for evaluating ethnographic work, which emphasise substantive contribution, reflexivity, impact and the expression of lived reality (Richardson 2000). Substantive contribution was supported by the first author's professional background as an intensive care nurse and by the triangulation of data generation methods. This allowed ventilation‐related decision work to be examined from multiple perspectives while remaining grounded in ICU practice. Reflexivity was addressed through continuous analytic and reflexive memo writing, regular discussions within the research team, and interdisciplinary research workshops. In addition, a large language model (ChatGPT, OpenAI) was used as a complementary reflexive aid to explore alternative formulations and perspectives on selected analytic ideas and conceptual relations. Its outputs were treated as stimuli for reflection, comparable to external inputs in analytic discussions. For this purpose, the tool did not have access to empirical data, and it was not used for coding, data analysis, or the development of analytical categories. All analytic decisions remained with the research team. Accordingly, the use of ChatGPT did not constitute an analytical method but served as a reflexive resource, complementing rather than replacing team‐based analytic discussions.

## 3 Findings

Analysis of observations and interviews identified the relational nature of two types of ventilation‐related decisions—directional and adjustment decisions—that structure decision‐making across the ICU trajectory. Both decision types are constituted through four interrelated domains of decision work: orienting, involving, negotiating and implementing. Findings are illustrated with excerpts from fieldnotes and interviews, translated from German into English.

### 3.1 Two Types of Ventilation Decisions

Our analysis indicates that ventilation‐related decisions in daily practice take two principal forms: directional and adjustment decisions. While closely connected in practice, they represent distinct forms of decision work, differing in their nature and in the focus of activities they require from ICU staff, yet forming a dynamic and interrelated structure of ventilation‐related decision work. Both types encompassed a wide range of variations, but the following sections present their representative forms as identified in the data.

#### 3.1.1 Directional Decisions: Setting the Course

By directional decisions we refer to decisions concerning the patient's ventilation within the overall illness trajectory. Typical examples of this type include questions of intubation, extubation, or the mode of planned weaning. These decisions set the overall course of ventilatory management by defining the intended direction of the patient's trajectory at specific points in time.

Directional decisions were often negotiated and determined within established structures such as ward rounds. These situations were characterised by their group setting and by access to electronic documentation systems, both of which allowed the retrieval of a wide range of information and typically involved some degree of discussion among involved clinicians. However, despite the frequent involvement of various actors and regular discussion, directional decisions were typically made by consultants (senior specialist physicians with supervisory responsibilities) when they were available. Residents, nurses and members of other professions, such as physiotherapists, occupational therapists, speech and language therapists and social workers usually contributed information to these processes and could, at times, also bring in their own ideas:

But at least when the senior consultants are present, we do not make the decisions. Still, we are heard, and if you have a good idea, it is accepted and implemented. (Interview 17, resident, early ICU experience)

The implementation of directional decisions was often processual and depended on a series of adjustment decisions through which the chosen course was enacted in practice.

#### 3.1.2 Adjustment Decisions: Nursing Autonomy When Aligning Patient and Technology

Adjustment decisions aim to achieve alignment between the patient and the ventilatory technology, oriented towards realising the directional decision. In doing so, they constitute the ongoing work through which directional decisions are translated into practice. Such alignment can be achieved through adjustments on the technological side, for example by modifying inspiratory pressure or selecting an appropriate mask for non‐invasive ventilation. It can also be established through patient‐related adjustments, such as modifying sedatives, providing guidance, or changing positioning. Hybrid forms that combine both approaches were likewise possible and were regularly observed in daily practice. This highlights that alignment was not limited to technical adjustments but involved multiple, interrelated forms of practices on both the patient and the ventilatory system.

Adjustment decisions were frequent and occurred mainly in the patient's room, sometimes within small group constellations, but typically by individual clinicians. Less experienced staff members, however, frequently sought confirmation or advice from more experienced colleagues before acting:

So if I'm unsure at first about any settings, or if I see that what I've done hasn't worked so well, I first ask my experienced colleagues or my shift leader (…). (Interview 8, nurse, early ICU experience)

Nurses played a central role in adjustment decisions and often exercised substantial autonomy in this area on the observed units. Accordingly, adjustment decisions were largely organised as bedside work, with nurses often taking primary responsibility for maintaining the alignment between patient and technology. However, interviewees reported that such autonomy was more limited on other ICUs where they had previously worked. When physicians were present in the room, this autonomy was often reconfigured. Temporarily, for example during rounds, physicians assumed control over the alignment of patient and technology, whether the nurse was present or not:

During the morning round, a consultant checks the ventilator, notes a very short inspiration time, and adjusts the ventilator without consulting the bedside nurse. (Observation 7)

This temporary shift in control occasionally caused frustration among nurses and gave rise to moments of interprofessional tension:

And it happens occasionally that during the midday round or the morning round, the ventilation mode is changed quickly without consulting the nurse. (…) Yes, that does happen, and it leads to irritation. (Interview 16, nurse, extensive ICU experience)

Such moments indicate that adjustment decisions were also sites of negotiation over professional roles and boundaries, even when no explicit discussion took place.

### 3.2 Four Domains of Work in Ventilation Decision‐Making

The work underlying both types of decisions was structured by four interrelated domains of practice: orienting, involving, negotiating and implementing. While presented separately here for analytical clarity, these domains were closely intertwined in practice and often overlapped and recurred within decision work.

#### 3.2.1 Orienting: Understanding the Situation

The first domain of decision work, orienting, referred to the gathering and interpretation of a broad range of information to form a picture of the patient's current condition and the wider care context. This included deliberate activities such as reviewing medical records, checking laboratory results or imaging and conducting physical examinations, as well as ongoing, often incidental, information gathering during bedside care. In practice, orienting extended beyond patient‐specific data and also encompassed organisational factors such as staff availability or bed capacity. It often involved two or more healthcare professionals, particularly nurses and physicians, during handovers, ward rounds, or informal exchanges and occasionally included conversations with patients and relatives. Orienting went beyond information gathering; it constituted the work through which the situation was defined in terms relevant for subsequent decisions.

Access to, and authority over, different types of information varied across professional groups, most prominently between nurses and physicians. This was not only a matter of perspective and expertise but was also shaped by the structural organisation of work. Nurses' prolonged bedside presence enabled continuous observation and direct assessment. Physicians, in contrast, were structurally positioned to gather and review diagnostic information, such as prior records, imaging and laboratory results, as part of their daily workflow. These arrangements influenced which information was noticed, prioritised and introduced into the decision‐making process. As one consultant noted:

Physicians of course have to assess the underlying condition. They (…) know the medical history better, (…), they know the lab results better (…). Nurses, on the other hand, gain more of a direct impression at the bedside. The contact times are simply completely different. (Interview 3, consultant, extensive ICU experience)

These differences shaped not only what information was available but also how the situation was framed and which aspects were foregrounded or remained peripheral in the decision work.

#### 3.2.2 Involving: Getting Into the Decision

The second domain of decision work, involving, describes how different actors enter the ventilation‐related decision work, whether through formal inclusion or their own initiative. Physicians and nurses were identified as central actors in our data, but other members of the interprofessional care team were also involved depending on the situation, as were, in some cases, patients. Table 2 illustrates four types of involving practices with extracts from the empirical data—structural, routine‐based, situational and non‐involvement.

**TABLE 2:** Types of involving practices.

Examples	Prospective	Retrospective
Structural	An interprofessional ward round takes place in which all participants are able to contribute. (Observation 16)	During a meeting, charge nurses are informed of decisions made by physicians, which they then relay to the bedside nurses. (Observation 13)
Routine‐based	“…then of course we should still go to the physician and say, ‘Hey, you, I think we could extubate him. Swallows, does this and that, everything's working’—just to get the ‘go‐ahead’ again accordingly.” (Interview 11, nurse, early ICU experience) “I think if a nurse extubated someone without consulting me, I wouldn't like it.” (Interview 17, resident, early ICU experience)	“So what I mean is, I actually adjust all the parameters, but if I had to immediately raise my FiO_2_ from 25 to 90% because the patient's respiratory status was just getting bad, then that's an immediate danger or an acute deterioration of the patient. And then I also let a doctor know.” (Interview 1, nurse, mid‐level ICU experience)
Situational	Patient is fighting the ventilator; nurse calls a physician and asks if she may administer a muscle relaxant. (Observation 7)	Patient is fighting the ventilator; nurse administers boluses of analgesic sedation already running via syringe pumps and informs the physician afterwards. (Observation 7)
Non‐involvement	A physician reduces flow and FiO_2_ for a patient on high‐flow therapy without informing the responsible nurse. When the patient subsequently reports shortness of breath, the nurse responsible for the patient is occupied elsewhere, requiring another nurse to intervene. (Observation 3)	

Involvement took different forms. Some were explicitly embedded in formal structures, such as ward rounds, where participation was predetermined by organisational design. In contrast, situational involvement occurred in response to specific circumstances, for example when additional team members were consulted because of their recognised expertise. Between these forms, our analysis identified patterns we describe as routine‐based involvement, referring to practices that are routine for clinicians familiar with the unit, often remain undocumented, and are sustained in everyday practice, outside formal structures. Involvement could also be differentiated by timing. Prospective involvement occurred when actors were present before or while a decision was being made, providing the opportunity to influence its content. Retrospective involvement, in contrast, referred to situations in which actors were only informed after a decision had already been taken. This temporal dimension of involvement influenced when actors entered the decision‐making process, including situations in which they became involved only after a decision had been established, yet could still intervene in its ongoing enactment. Moreover, we explored types of non‐involvement that proved relevant for interprofessional interaction, sometimes carrying the potential for and highlighting tensions around inclusion and exclusion in interprofessional decision work. Involvement describes who takes part in the decision‐making process. It does not, however, automatically translate into influence on the negotiation of the decision (see next domain).

#### 3.2.3 Negotiating: Struggling for a Decision

The negotiation domain focuses on how decisions are established in practice, including both explicit negotiation and forms of non‐negotiation. Negotiation practices included argument‐based negotiation, persuasive negotiation and action‐based negotiation. Non‐negotiation practices were identified as non‐negotiation by silence and non‐negotiation by implementation. Together, these practices illustrate that decision‐making was not only shaped through explicit negotiation but also through forms of non‐negotiation that structured how and whether decisions were contested. The following sections describe each in more detail, with examples from observations and interviews.

Argument‐based negotiation was not only a frequent practice, but also consistently valued as constructive by interview participants. Here, argument is understood as the exchange of reasons and justifications, not as interpersonal conflict. In this form, actors advanced fact‐based arguments, drawing on laboratory or monitoring data, prior interventions, clinical experience and bedside assessments. Such exchanges were often embedded in established structures but could also occur in small, ad hoc constellations:

Two ICU nurses and a senior consultant stand in front of the ventilator, discussing the patient's lack of spontaneous breathing efforts. (Observation 7)

These exchanges illustrate how clinical reasoning was made explicit and used to justify and stabilise decisions within interprofessional interaction. We also observed persuasive negotiation, aimed at steering the decision towards one's preferred course. This could involve making one's emotions or burdens explicit, or using deliberate exaggeration:

During a detailed discussion about extubation in a particularly sensitive case, a resident who had previously expressed dissatisfaction with the chosen course uses deliberate exaggeration to signal frustration, stating: 'He's 20—let's just let him die, whatever.'(Observation 5)

Such practices indicate that negotiation was not limited to fact‐based reasoning but also involved affective and strategic elements aimed at shaping the direction of decisions. Distinct from these verbal negotiation practices, action‐based negotiation involved influencing the decision by acting or by deliberately omitting an action, as illustrated in the following account by a resident:

Another colleague (…) had already told the nurses, ‘Yes, we have to intubate him. The family wants this, there is no clear limitation of therapy, and we cannot let him die from CO_2_ narcosis.’ (…) And then there was quite a lot of resistance from the nursing staff because they simply did not see enough potential benefit. I mean, the patient only lived for another week, and I understand that. But there was very little understanding at the time. They even refused to prepare for the intubation. (Interview 7, resident, mid‐level ICU experience)

In these cases, actions themselves became a means of negotiation, allowing actors to influence decisions in ways that could outweigh verbal disagreement.

In addition to explicit negotiation practices, our analysis identified forms of non‐negotiation that played a central role in how decisions were established in practice. These practices did not indicate an absence of engagement but represented distinct ways in which decisions were stabilised or left uncontested. Among non‐negotiation practices, non‐negotiation by silence occurred when actors refrained from actively engaging in the decision‐making process. Such silence could reflect hierarchical constraints but could also express trust in the expertise of those actively negotiating:

If, for example, there is already consensus between the nurse in the room, who is experienced, and our consultants—I honestly rarely feel, given my own level of experience, that I would disagree or be unable to support the decision. (Interview 13, resident, mid‐level experience)

In these situations, silence functioned as a form of participation that shaped the decision‐making process by leaving existing positions unchallenged. Non‐negotiation by silence was also seen when actors were involved but unable or unwilling to actively contribute to decision work.

A nurse waits outside the isolation room for the ward round to reach the patient he is responsible for. When the senior consultant arrives and asks, ‘What's going on with the patient?’, the nurse replies, ‘Nothing,’ and no further exchange follows. (Observation 2)

Such instances illustrate how non‐negotiation could limit the articulation of alternative perspectives, even when actors were formally involved. In contrast to action‐based negotiation, where actions were used strategically to influence an ongoing discussion, non‐negotiation by implementation referred to situations in which an irreversible act effectively closed the space for further negotiation.

A consultant, referring to documented ventilation parameters and under pressure due to bed shortages, proceeded to extubate a patient without prior consultation with another consultant. The latter later reacted with irritation at not having been involved. (Observation 4)

This form of non‐negotiation shifted decision work from discussion to action, effectively resolving the decision through practice.

#### 3.2.4 Implementing: Getting Things Done

The last domain of decision work refers to implementing the chosen decision. However, implementation did not simply follow decisions but constituted an integral part of decision work, as actions taken in practice could reshape or redefine the chosen course. Practices in this domain involved a wide range of actions, from technical procedures such as intubation, extubation, or adjusting ventilator settings, to patient‐related measures such as mobilisation and positioning. While implementing practices could mark the conclusion of a given decision‐making process, they often represented only a transition point that generated further decision work:

During non‐invasive ventilation, air is not reaching a patient's lungs effectively. After short‐lived improvements with positioning, the resident inserts a nasopharyngeal airway, yet ventilation remains insufficient and further steps are considered. (Observation 3)

This illustrates how implementation could directly generate new decision‐making situations, requiring ongoing adjustment and reassessment. In some situations, implementation did not simply realise a previously agreed directional decision but modified it in practice. Such instances demonstrate that decisions could be reshaped during their implementation, even without explicit renegotiation.

During handover, the early shift nurse told his colleague that he did not consider the patient ready for extubation and that he had not performed the ‘strange test', referring to the spontaneous breathing trial. Later, the omitted trial became a source of irritation during the physicians' handover. (Observation 5)

Such instances did not necessarily involve open disagreement but nevertheless altered the course of ventilatory management in ways that were not explicitly negotiated.

### 3.3 A Conceptual Framework of Ventilation‐Related Decision Work in ICUs

The above findings can be brought together as a conceptual framework of ventilation‐related decision work in ICUs. Figure 1 visualises the relationships between the central elements of this framework.

**FIGURE 1:**
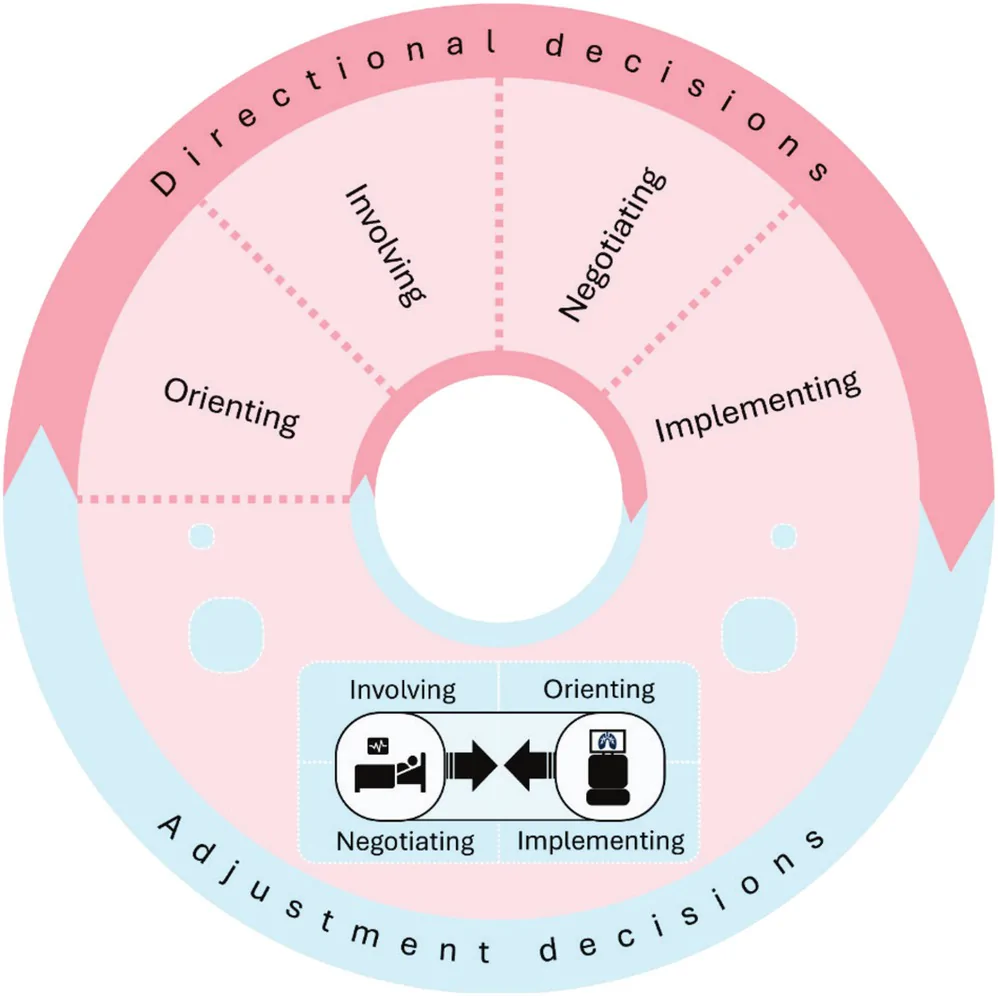
Relational organisation of ventilation‐related decision work in ICUs.

Our framework conceptualises ventilation‐related decision work as a continuous process unfolding along the patient's critical illness trajectory. This process is constituted through two interrelated types of decisions: directional and adjustment decisions. Typically, multiple adjustment decisions serve to implement directional decisions in a processual manner by aligning patient and ventilation support systems. However, decision work does not unfold linearly. Adjustment decisions can reshape directional decisions when their outcomes require a re‐evaluation of the previously chosen course or reveal that the intended course cannot be achieved. Figure 1 visualises this recursive relationship by depicting adjustment decisions as embedded within the implementation of directional decisions. The upward trajectory of the blue arrow indicates that adjustment decisions may recursively influence subsequent directional decisions. Both directional and adjustment decisions are accomplished through practices across all domains, rather than being tied to specific domains. The aforementioned circularity also applies to the four domains through which both decision types can be analytically described. For example, while orienting may serve as a starting point for a particular decision process, practices within this domain also accompany and shape involving, negotiation and implementation. Activities in the domain of implementing may require involving experts and thus result in new negotiations. Likewise, work in the domains of directional decisions can shape adjustment decisions and vice versa—for example when practices of implementing an adjustment decision simultaneously contribute to the negotiation of a directional decision. At the same time, not all domains are equally relevant in every episode of decision work. Some situations may unfold without explicit practices of involving or negotiating, as in routine adjustment decisions such as the reduction of FiO_2_, which are often enacted without broader interprofessional engagement. This variability underscores that the domains represent analytical distinctions and do not constitute fixed or sequential stages of decision‐making. They offer overlapping analytical perspectives on decision work, through which individual practices may simultaneously relate to multiple domains. For example, implementation can also constitute a form of non‐negotiation, effectively closing spaces for further deliberation.

## 4 Discussion

This study aimed to conceptualise how ventilation‐related decisions are accomplished in interprofessional ICU practice. Building on an analytical perspective that understands decision‐making as ongoing decision work, our analysis shows how decisions are accomplished as a socially organised form of work unfolding across the illness trajectory. The following sections discuss four key aspects that become visible through this perspective: the temporal and iterative organisation of decision work, involving the organisation of participation and access to influence, the role of non‐negotiation in accomplishing decisions and implementation as a constitutive and relational dimension of decision work.

### 4.1 The Temporal and Iterative Organisation of Decision Work Across the Illness Trajectory

Conceptualising ventilation‐related decision‐making as temporally organised decision work shifts attention away from discrete decision points or the selection of a preferred course of action (e.g., Banning 2008; DeKeyser Ganz et al. 2016; Tiffen et al. 2014) towards the ongoing accomplishment of decisions across the illness trajectory. This shift in perspective opens up several analytical possibilities. In particular, it makes visible how adjustment decisions do not merely enact directional decisions but can actively reshape them, as ongoing efforts to align patient and technology feed back into the projected course of action. While this distinction may resemble familiar differentiations between strategic, tactical and operational decision‐making, our analysis does not describe different levels of decision‐making. Instead, it captures different forms of work that are closely intertwined in practice. In this sense, directional decisions contribute to structuring the trajectory by defining a provisional course of action, while adjustment decisions form part of the ongoing arc of work through which this trajectory is continuously enacted and revised (Strauss et al. 1997). As part of this arc, adjustment decisions are not isolated decision processes but embedded in continuous efforts to align patient condition, technological support and clinical goals over time. Our conceptualisation of adjustment decisions resonates with Strauss' notion of machine work, particularly the category of connecting work (Strauss et al. 1997). This refers to practices through which connections between bodies and machines are established and maintained in the course of care, constituting a distinct form of work that varies with the patient's condition and trajectory phase. This highlights that a substantial part of ventilation‐related decision work consists of ongoing efforts to align patient and technology, rather than discrete acts of choosing between predefined options. Approaches that conceptualise decision‐making primarily as cognitive or deliberative processes may not fully capture these forms of work. Moreover, these practices do not remain confined to a single domain. Efforts to align patient and technology, as described in connecting work, can feed back into orienting by generating new information about the patient's condition and the viability of the projected course of action. Decision work thus unfolds through recursive movements across domains, not as a linear progression.

### 4.2 Involving as Organising Participation and Access to Influence in Decision Work

While frameworks such as collaborative clinical reasoning (Kiesewetter et al. 2017) or interprofessional shared decision‐making (DeKeyser Ganz et al. 2016) often treat participation as a given, conceptualising involving as a domain of decision work foregrounds how participation is actively organised in practice and thus shapes decisions. This aligns with previous work showing that nurses often need to create opportunities to contribute and strategically position their knowledge to gain influence within interprofessional decision‐making processes (Caronia et al. 2020; Manias and Street 2001). The domain of involving is closely intertwined with negotiated order, as it both reflects and contributes to the organisation of interprofessional relations. Actors who are not involved at specific moments cannot participate in negotiating decisions, highlighting that involvement constitutes a condition for influence within decision work. Patterns of involvement therefore also shape who is able to contribute to the negotiated order more broadly, as repeated non‐involvement limits opportunities to actively shape interprofessional relations. At the same time, our findings suggest that not only whether actors are involved matters, but also how and when they are involved. A recurrent pattern was the retrospective involvement of nurses, particularly in the context of implementation, where they were brought into decision work after directional decisions had already been set. These forms of retrospective involvement become visible when decision‐making is analysed as ongoing decision work, not as discrete decision moments in which participation would appear as given. The domain of involving offers a way to account for the social complexity of decision‐making in interprofessional care. It opens analytical space to examine how participation is continuously organised and reconfigured across situations. In this sense, interprofessional decision work is not characterised by fixed patterns of collaboration but by shifting constellations of involvement, in which actors are selectively brought into or kept outside of particular decision situations, and through which interprofessional relations are continuously organised as negotiated order.

Although patients were rarely involved as active participants in the ventilation decisions analysed here, their condition and, at times, preferences remained a central reference point in decision work. Importantly, patient participation is a normative goal of contemporary intensive care (Secunda and Kruser 2022), and the literature suggests that participation in intensive care extends beyond direct treatment decisions and encompasses varying forms of engagement depending on patients' abilities and preferences (Falk et al. 2019; Slettmyr et al. 2022). At the same time, the role of relatives in intensive care decision‐making has been discussed more widely (Olding et al. 2016), especially in broader discussions of shared decision‐making, particularly in relation to far‐reaching decisions such as end‐of‐life care (Michalsen et al. 2019). Conceptualising involving as a domain of decision work extends the analytical lens beyond healthcare professionals by making visible not only how patients and families are included in decision‐making, but also how their non‐involvement is accomplished through everyday practice. In situations or settings where patients are able to participate more actively, the same domain could capture how decision work is redistributed between professionals, patients and families, thereby shaping both interprofessional collaboration and the nature of shared decision‐making. In addition, this perspective allows consideration of non‐human actors within decision work. Intensive care units are highly technological environments, in which a wide range of devices are integral to everyday clinical practice (Almerud et al. 2008; Wikström and Sätterlund Larsson 2003). It is reasonable to assume that these technologies are not neutral but shape interprofessional decision‐making, even though this relationship has rarely been examined explicitly (Küpper, Schmucker, Elsbernd, et al. 2026).

### 4.3 The Role of Non‐Negotiation in Accomplishing Decision Work

Part of our conceptualisation of decision work is the domain of negotiation. While forms of content‐focused deliberation are well captured in approaches such as collaborative clinical reasoning (Kiesewetter et al. 2017) or distributed cognition (Wilson et al. 2022), attending to practices of non‐negotiation opens up additional analytical perspectives. Although such instances have been noted in previous studies on interprofessional collaboration (Reeves et al. 2009), our analysis integrates them into a framework of decision work and specifies their role in the accomplishment of decisions. This makes it possible to grasp absences within processes of negotiation and to examine how decisions are accomplished without explicit deliberation. Our analysis suggests that such non‐negotiation takes different forms and is embedded in distinct practical logics. In some situations, the absence of negotiation appeared to reflect trust in the expertise of those actively engaged in decision work. In others, it functioned to avoid or limit discussion, for example when potential disagreement was not taken up. The domain of negotiating thus extends existing work by accounting for situations in which actors are involved but do not actively engage in negotiation. Practices of non‐negotiation, such as silence or implementation without prior discussion or in deviation from previously agreed decisions, shaped decisions by effectively closing spaces for contestation. Negotiated order in ventilation‐related decision work is thus not only produced through explicit negotiation but also through such practices of non‐negotiation. These do not indicate an absence of engagement but constitute a mechanism through which boundaries of influence and responsibility between professional groups are stabilised, including the delineation of what becomes non‐negotiable in a given situation. This perspective also links decision work to patient safety, as practices of non‐negotiation may reduce opportunities for speaking up, which is considered essential for preventing adverse events (Kane et al. 2023).

### 4.4 Implementation as a Constitutive and Relational Dimension of Decision Work

Clinical decision‐making models often conceptualise decision‐making as culminating in a choice, with implementation treated as a subsequent phase (DeKeyser Ganz et al. 2016; Tiffen et al. 2014). In contrast, our findings indicate that implementing is not merely a downstream activity but a constitutive dimension of decision work. This becomes particularly visible in the relationship between directional and adjustment decisions. While directional decisions define a provisional course of action, their realisation depends on adjustment decisions, which are enacted through practices of implementing. Implementing thus constitutes a key mechanism through which adjustment decisions both realise and potentially reshape directional decisions. More fundamentally, such constitutive relations between action and decision remain difficult to capture within predominantly cognitive conceptualisations of clinical decision‐making, which primarily seek to explain how decisions are formed rather than how they are accomplished in practice (Nibbelink and Brewer 2018). Implementation also operates as a relational dimension of decision work, linking different forms of decision‐making across time and practice. Importantly, implementation also creates opportunities for interprofessional influence that are not captured in models focusing on formal decision authority. In particular, bedside nurses play a central role in implementing ventilation‐related decisions through their continuous involvement in patient care. Through practices of implementing, which are closely intertwined with other domains such as action‐based negotiation and non‐negotiation by implementation, they are able to influence directional decisions that may formally appear to be dominated by physicians. These forms of practice do not rely on explicit negotiation but nevertheless shape the stabilisation or modification of previously defined courses of action. The extent to which such practices are accepted appears to depend on the unit's culture and other contextual factors shaping ventilation‐related decision work (Küpper, Schmucker, Hahn, et al. 2026).

#### 4.4.1 Strengths and Limitations

This study offers an in‐depth, ethnographically grounded conceptualisation of ventilation‐related decision work in two ICUs within a single healthcare system. A key strength lies in its ethnographic design, which enabled analysis of decision work as it unfolds in practice. In addition, the study develops a conceptually grounded framework that extends existing approaches to clinical decision‐making by focusing on the organisation of decision work. As such, the findings are not intended to be empirically representative of international ICU settings. Their transferability lies at the conceptual level, providing analytic distinctions and sensitising concepts that may support the examination of decision work in other contexts. The analysis concentrates on the organisation of ventilation‐related decision work in ICUs and therefore primarily attends to interactional and practical dimensions of decision‐making. While this study adopts an interprofessional perspective, interview data were limited to nurses and physicians, rendering other professional perspectives less analytically visible. However, the proposed conceptualisation of decision work is not profession‐specific. It provides an analytical framework for examining how decision work is organised across different professional constellations. Finally, as with all ethnographic research, the analysis reflects interpretative work shaped by the researchers' positionality and sustained engagement with the field. While this limits claims of neutrality, it supports a context‐sensitive understanding of how ventilation‐related decision work is organised in everyday ICU practice.

#### 4.4.2 Implications for Research

The conceptualisation of decision work developed in this study opens several avenues for future research. It may be applied to other clinical contexts to examine how decision‐making is organised across different settings, professional constellations and organisational conditions. While the present analysis foregrounds the temporal organisation of decision work, spatial aspects were also evident in the data, for example in shifts of nursing autonomy when senior physicians entered the patient's room, but were beyond the scope of the present analysis. These observations suggest that space may constitute an additional analytical dimension of decision work, influencing how and where negotiation and non‐negotiation unfold. Given that spatial dimensions remain under‐conceptualised in interprofessional research (Kitto et al. 2013), further work could usefully examine how spatial arrangements intersect with temporal processes in the accomplishment of clinical decisions. Future research could also extend the analysis to include patients, relatives and non‐human actors more explicitly, in order to further develop an understanding of decision‐making in complex care environments.

#### 4.4.3 Implications for Policy and Practice

Understanding ventilation‐related decision‐making as ongoing decision work highlights the need to recognise and support the distributed organisation of decision processes in intensive care. This includes making visible and legitimising the role of nurses in shaping decisions through ongoing adjustment and implementation practices. At an organisational level, this perspective may inform how interprofessional collaboration and decision‐making responsibilities are structured in complex care settings.

## 5 Conclusion

Ventilation in the ICU is not the result of a singular decision—it is worked out over time, involving human and non‐human actors. This study conceptualises ventilation‐related decision‐making as ongoing interprofessional decision work. By differentiating directional and adjustment decisions and analysing how orienting, involving, negotiating, and implementing intertwine, it provides a framework for understanding how decisions are accomplished in practice. This perspective makes visible how decisions are shaped through ongoing interaction, including through implementation, and brings into view contributions of nurses that would remain outside the scope of conventional decision‐making models. It thus offers a practice‐oriented approach to decision‐making in complex, interprofessional and technology‐intensive care settings, with potential relevance beyond the ICU.

## Author Contributions

A.K., M.S., A.E., S.K. and C.M. jointly developed the study concept. The methodological framework was designed by A.K. and C.M. Validation was performed collaboratively by A.K., M.S., L.H., A.E., S.K. and C.M. Formal data analysis was undertaken by A.K., M.S. and L.H. A.K. led the data collection, visualisation and preparation of the original manuscript draft. All authors (A.K., M.S., L.H., A.E., S.K., C.M.) contributed to critical review and revision of the manuscript. A.E., S.K. and C.M. provided supervision throughout the study. Project administration was managed by A.K. and C.M. and funding was secured by A.E. and C.M.

## Generative‐AI‐Disclosure

Generative AI tools were used during the preparation of this manuscript to support specific stages of the research and writing process. The software aTrain was employed for transcribing interview recordings. DeepL and ChatGPT were used to translate selected excerpts from interviews and field notes from German into English. ChatGPT also supported clarity, language and style. All AI‐assisted outputs were carefully reviewed and edited by the authors, who take full responsibility for the content and the final version of the manuscript.

## Funding

This study is part of a doctoral project supported by the Federal Ministry for Research, Technology and Space (BMFTR), grant number 03FHP115. The funding agency had no involvement in the study design, data collection, analysis, interpretation, or writing of the manuscript.

## Disclosure

Given the conceptual nature of the study and the need to adequately situate and distinguish the proposed framework within existing literature on clinical decision‐making, the reference list slightly exceeds the recommended limit.

## Ethics Statement

The study was conducted in accordance with the principles of the Declaration of Helsinki. Ethical approval was granted by the Ethics Committee of the University Hospital Tübingen (project number 142/2023B02).

## Conflicts of Interest

The authors declare no conflicts of interest.

## Data Availability

The data that support the findings of this study are available on request from the corresponding author. The data are not publicly available due to privacy or ethical restrictions.
